# Supplementary material for: Reversible and controllable reduction in friction of atomically thin two-dimensional materials through high-stress pre-rubbing
Source: Nat Commun. 2024 Nov 15;15:9897. doi: 10.1038/s41467-024-54363-2 (PMC11568223; doi:10.1038/s41467-024-54363-2)
Supplement: Supplementary file 1 — Supplementary Information [file 41467_2024_54363_MOESM1_ESM.pdf]

SUPPLEMENTARY INFORMATION

TITLE

Reversible and Controllable Reduction in Friction of Atomically Thin Two-Dimensional Materials through High-Stress Pre-Rubbing

AUTHORS

Haoyang Su[1#], Honglin Zhang[2#], Junhui Sun[2#], Haojie Lang[1], Kun Zou[1], Yitian Peng[1]*

[1]College of Mechanical Engineering, Donghua University, Shanghai 201620, China.

[2]Tribology Research Institute, State Key Laboratory of Traction Power, School of Mechanical Engineering, Southwest Jiaotong University, Chengdu 610031, China.

[*]Email: yitianpeng@dhu.edu.cn

These authors contributed equally: Author Su HY, Author Zhang HL, and Author Sun JH.

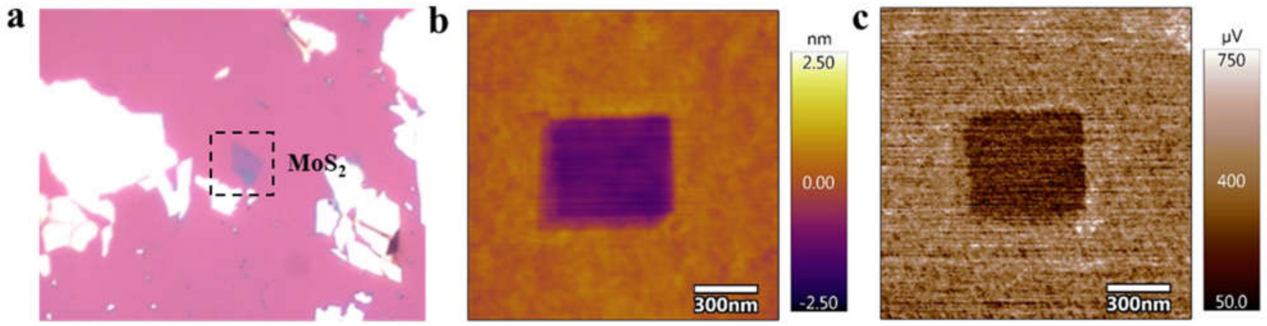

Supplementary Figure 1. Pre-rubbing and friction measurements of MoS_2 on SiO_2/Si substrate. (a) Optical image of a 1.1nm thick MoS_2 flake deposited on SiO_2/Si substrate. (b) Topography image after pre-rubbing under 10.0 GPa, note that a drift occurs during the scanning-down stage. (c) Friction mapping after pre-rubbing (with a normal load of 50nN) acquired simultaneously with topography.

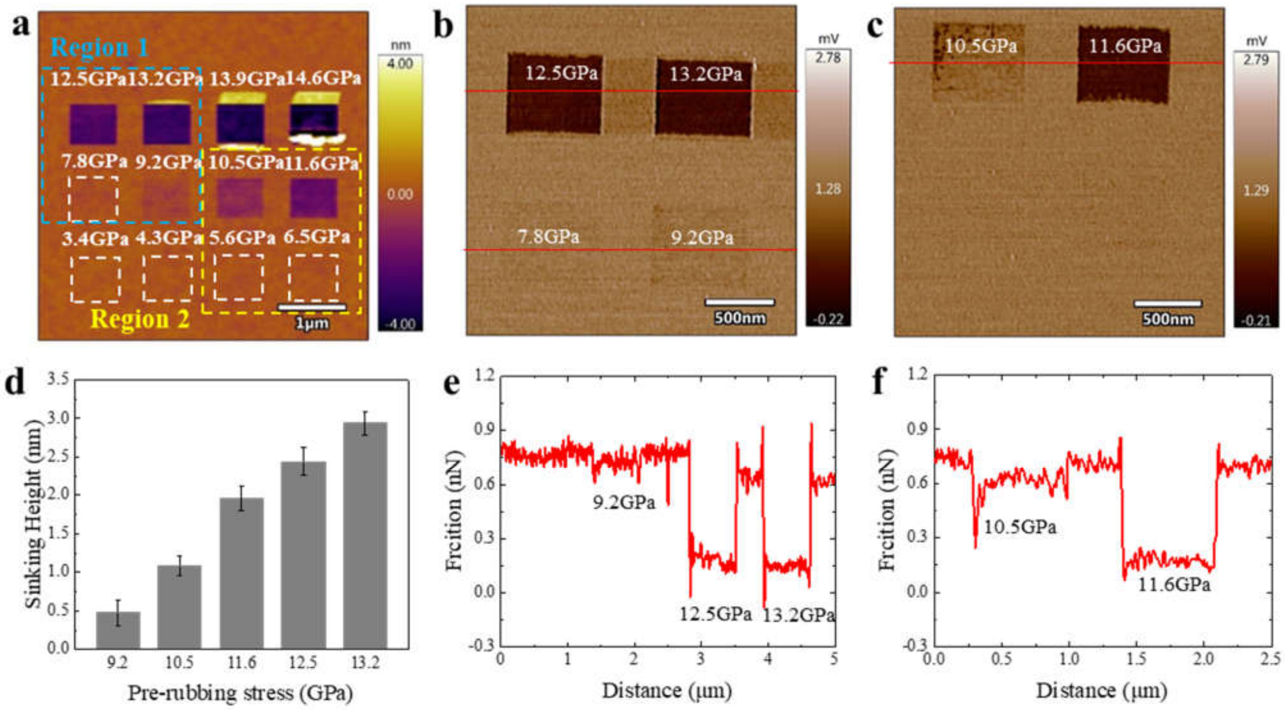

Supplementary Figure 2. Friction mapping of the graphene pre-rubbed under different stress levels. Acquired after 10 days, it involves localized scanning to avoid damaged areas, using a Si tip (Multi75Al-G, 3.0 N/m, Budget Sensors). (a) Topography image acquired in noncontact mode. (b) and (c) presented the friction mappings of Region 1 and Region 2 as illustrated in (a) at a normal load of 20nN, respectively. (d) Average sinking height as a function of the pre-rubbing stress, the error bars represent the standard deviation of the data. (e) and (f) depicted the profiles corresponding to the red lines in (b) and (c) respectively. Note that the two traces in (b) have been linked in (d).

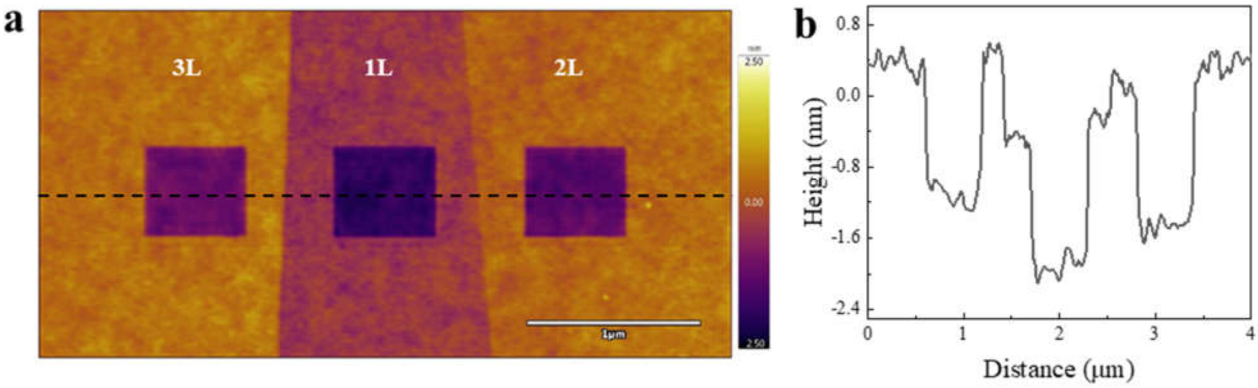

Supplementary Figure 3. Pre-rubbing experiments on the distinct thickness of graphene. (a) and (b) demonstrate the morphology and cross-sectional data profiles along the black dashed lines.

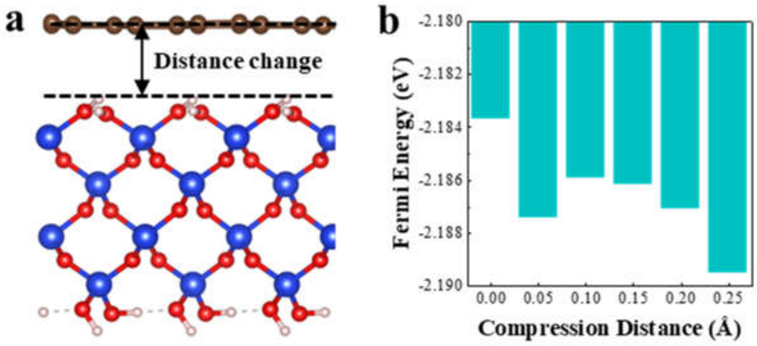

Supplementary Figure 4. Fermi level calculation in DFT. (a) Schematic diagram of the model. (b) The relationship between Fermi level and different compression distances.

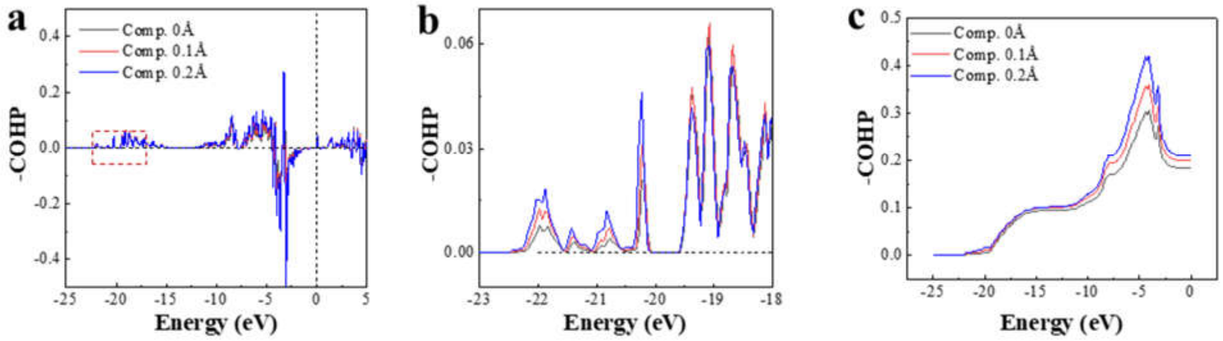

Supplementary Figure 5. Crystal orbital Hamiltonian population (COHP). (a-b) Crystal orbitals Hamiltonian population (COHP) under different compression conditions, (c) COHP integral diagram under different compression distance.

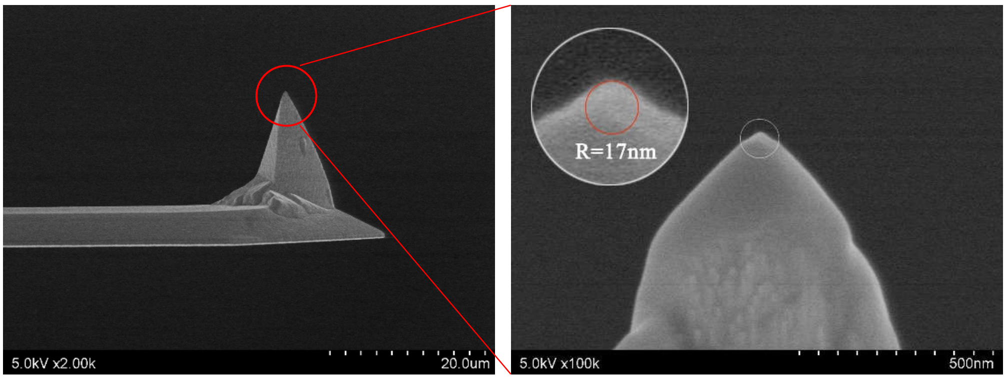

Supplementary Figure 6. The characterization of the tip. SEM images of the probe and an estimation of the tip radius after pre-rubbing.
