# Supplementary material for: Reversible and controllable reduction in friction of atomically thin two-dimensional materials through high-stress pre-rubbing
Source: Nat Commun. 2024 Nov 15;15:9897. doi: 10.1038/s41467-024-54363-2 (PMC11568223; doi:10.1038/s41467-024-54363-2)
Supplement: Supplementary file 2 — Description of Additional Supplementary Files [file 41467_2024_54363_MOESM2_ESM.pdf]

Description of Additional Supplementary Files

File Name: Supplementary Data 1
Description: Atomic coordinates used for density functional theory (DFT) calculations.
